# Study on Porosity Defect Detection in Narrow Gap Laser Welding Based on Spectral Diagnosis

**DOI:** 10.3390/ma16144989

**Published:** 2023-07-13

**Authors:** Jinping Liu, Baoping Xu, Yingchao Feng, Peng Chen, Cancan Yan, Zhuyuan Li, Kaisong Yang, Kun She, Yiming Huang

**Affiliations:** 1 China Nuclear Industry 23 Construction Co., Ltd., Nuclear Industry Research and Engineering Co., Ltd., China National Nuclear Corporation Key Laboratory of High Efficiency Welding, Beijing 101300, China; jinpingliu1990@163.com (J.L.); chenpeng23cn@163.com (P.C.);; 2 Fujian Fuqing Nuclear Power Co., Ltd., Fuzhou 350318, China; xubp@cnnp.com.cn; 3 Tianjin Key Laboratory of Advanced Joining Technology, School of Materials Science and Engineering, Tianjin University, Tianjin 300350, China; 4 School of Electrical and Information Engineering, Tianjin University, Tianjin 300072, China

**Keywords:** narrow gap laser welding, spectral diagnosis, laser-induced plasma, porosity defect

## Abstract

As an advanced connection technology for large thick-walled components, narrow gap laser welding has the advantages of small heat input and high efficiency and quality. However, porosity defects are prone to occur inside the weld due to the complex welding environment. In this study, the influence of the process parameters and pollutants such as water and oil on the porosity defect were explored. The action mechanism of water on the electron temperature and spectral intensity of the laser-induced plasma was analyzed. The results showed that the spectral intensity during narrow gap laser welding was weaker than that of flat plate butt welding. Under the optimal welding process conditions, the electron temperature during narrow gap laser self-fusion welding was calculated as 7413.3 K by the Boltzmann plot method. The electron density was 5.6714 × 10^15^ cm^−3^, conforming to the thermodynamic equilibrium state. With six groups of self-fusion welding parameters, only sporadic porosity defects were observed according to the X-ray detection. When there was water on the base metal surface, a large number of dense pores were observed on the weld surface and in the weld through X-ray inspection. Compared with the spectral data obtained under the normal process, the relative light intensity of the spectrometer in the whole band was reduced. The electron temperature decreased to the range of 6900 to 7200 K, while the electron density increased. The spectrum variation during narrow gap laser wire filling welding was basically the same as that of laser self-fusion welding. The porosity defects caused by water and oil pollutants in the laser welding could be effectively identified based on the intensity of the Fe I spectral lines.

## 1. Introduction

Large thick-walled components have been widely used in nuclear power construction, requiring high connection quality due to the unique high service environment. The narrow gap welding process can not only greatly reduce the groove filling area and improve the welding efficiency, but also can reduce welding deformation and residual stress, which is the main method for connecting large thick-walled components [1,2,3]. Traditional narrow gap gas tungsten arc welding produces a large heat input, which makes the grain coarse and the mechanical properties of the weld weakened. In addition, the movement range of the tungsten electrode at the root of the groove is small, and problems such as tungsten sticking and sidewall arcing are prone to occur [4]. Compared with the arc welding process, laser welding is characterized by concentrated energy density, small heat input, and high efficiency [5,6]. The narrow gap laser welding (NGLW) process can effectively improve the welding efficiency, refine the weld grain, and reduce the joint deformation and residual stress, meeting the welding requirements of low stress and high quality for large thick-walled components [7,8].

Compared with the laser welding process, the NGLW process is more complex and easily generates sidewall incomplete fusion, porosity, and so on. Using computer fluid dynamic simulation, Gu et al. [9] investigated the impact of laser beam reflection on incomplete fusion and microstructure evolution and classified the influence of laser beam reflection in the multi-pass NGLW process. Through a large number of experiments, Long et al. [10] found that the key to solve the incomplete fusion was the ratio of the welding line energy to the wire feed. Wang et al. [11] reduced the porosity defects effectively with ultrasonic assistance and found that the electron temperature and density of the laser-induced plasma increased [12]. Jiang et al. [13] found that laser oscillation brought a strong stirring effect, which changed the growth pattern of the sidewall grain and reduced the porosity defects.

However, there may be oil and water pollutants on the surface of the base material due to the complex on-site welding environment, resulting in porosity defects in the weld and serious damage to the strength of the weld joint [14,15]. Post-welding nondestructive testing technologies such as X-ray detection and ultrasonic inspection can effectively detect the internal defects of welds, but the test results are subject to the component size and the subjective judgment of inspectors [16]. In order to realize real-time detection of defects, acoustic emission, infrared photography, visual imaging, and other sensing means have been used to monitor the laser welding process [17,18]. Will et al. [19] used optical coherence tomography (OCT) to conduct online monitoring of the keyhole state during laser welding. Based on OCT, defect types were judged in real-time since the stability of the keyhole was strongly correlated with the generation of defects. Using a high-speed camera to collect images of the keyhole and plasma plume, Huang et al. [20] obtained features such as the keyhole area through image processing and established a one-dimensional convolutional neural network prediction model, which identified surface welding defects with a high accuracy. Zhao et al. and Huang et al. from Tianjin University [21,22] realized the online diagnosis of welding modes and surface defect types based on laser-induced plasma electrical signals. Unfortunately, constrained by the small observation view caused by the narrow gap, it was difficult to apply these sensor technologies in narrow gap laser welding.

Compared with the above sensors, spectral diagnosis technology has the unique advantage of obtaining the elemental information and thermomechanical properties of laser-induced plasma, which can reveal the dynamic interaction mechanism between the laser, materials, and plasma [23,24]. Through laser-induced breakdown spectroscopy technology, Lednev et al. [25] carried out online measurement in the laser welding of a nickel-chromium-based superalloy. It was found that the intensity of the iron and chromium atoms’ spectral lines increased abnormally when there were defects in the weld. In the laser deep penetration welding process, Li et al. [26] observed that the spectral intensity decreased with the increase of the penetration depth and used this as the input of a neural network to identify the penetration state. Huang et al. [27] found that there was a strong correlation between the H I spectral lines and porosity defects during the tungsten inert gas welding of aluminum alloys. It can be seen that spectral diagnosis technology has a good application promise in determining the welding quality. Therefore, this study intended to deeply explore the plasma characteristics of narrow gap laser welding by spectral diagnostic technology and clarify the influence of pollutants on the base metal surface on the plasma and weld internal quality.

## 2. Material and Experimental Procedures

**Figure 1:**
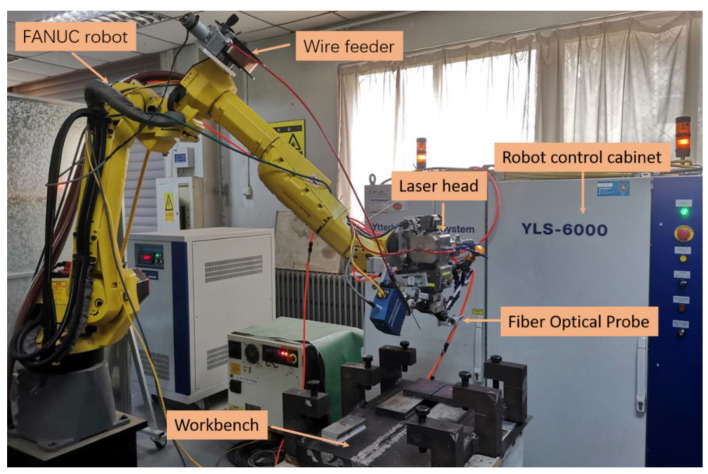
Experimental system.

**Figure 2:**
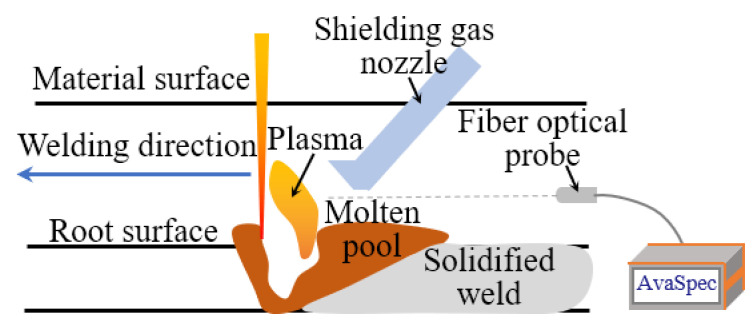
Spectral acquisition diagram.

The welding platform was composed of a robot, the laser equipment, and a sensor subsystem, as shown in Figure 1. The robot model was FANUC M-10i A, derived by the R-30iBA control cabinet. The robot has an arm span of 1422 mm and a load of 10 kg. The wire-feeding device adopts a DC motor for wire feeding. The wire-feeding speed ranges from 2 to 15 m/min. It is suitable for 0.6/0.8/1.0 mm wire feeding. The laser equipment model was YLS-6000-S2T-Y16 with a maximum laser power of 6 kW and a laser wavelength of 1070 nm. The beam parameter product was 5.9 mm*mrad, and the focal length was 200 mm. Due to the high stability performance and spot quality, it could realize the narrow gap multi-layer and multi-pass welding of large thick plates. According to the atomic spectrum database, the wavelengths of iron atoms are mainly concentrated in the band of 300–450 nm. Therefore, a AvaSpec-ULS4096CL-EVO spectrometer with a band of 220–487 nm and a resolution of 0.18 nm was selected in this study. In order to ensure that the spectral signal was not affected by the acquisition distance, a special fixture was designed to fix the optical fiber probe on the robot. When collecting spectral signals, the optical fiber probe was placed horizontally, as shown in Figure 2. The center line of the optical fiber was 5 mm above the surface of the molten pool, and the integrating time of the spectrometer was set at 20 ms.

**Figure 3:**
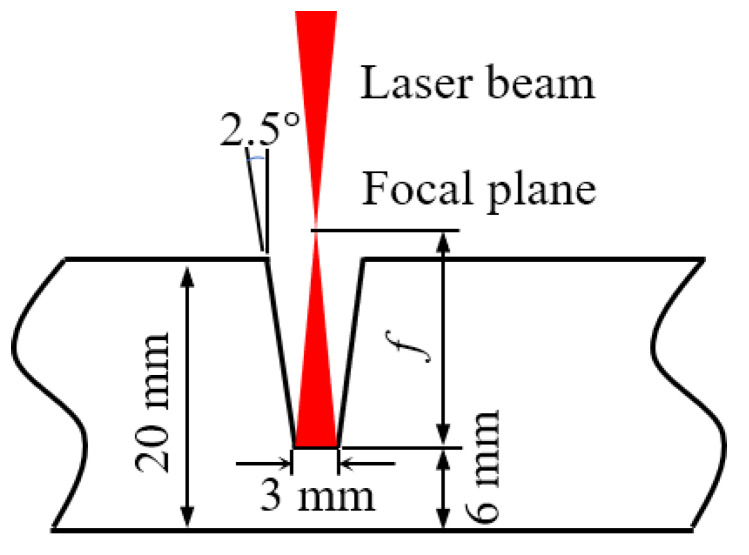
Groove design diagram.

The base material was A36 carbon steel of 250 mm × 100 mm × 20 mm, and the composition is shown in Table 1. The design of the groove shape is shown in Figure 3. The root height of the groove was 6 mm, and the groove angle was 5°. For the laser self-fusion welding experiments, the laser power was set at 4.5 kW, and other parameters are shown in Table 2. The focal plane was above the workpiece, with a value of 10 mm. The experiments were conducted to explore the influence of welding speed and shielding gas flow on weld forming and spectral data. Each set of tests in Table 2 was repeated twice. Laser wire filling welding was performed on the basis of appropriate self-fusion welding. The welding power was maintained as 4.5 kW during wire filling welding. Besides, the welding speed was set at 8 mm/s and the wire-feeding speed at 7.5 m/min. Laser wire filling welding was performed 4 times. In addition, in order to clarify the influence of pollutants (oil, water) on the weld quality under complex working conditions, pollutants were preset on the groove or previous weld surface as a comparative test. The experiments of laser self-fusion and laser wire filling welding with pollutants were repeated in 4 groups, respectively.

The electron temperature and electron density were the main parameters to characterize the thermodynamic properties of the laser-induced plasma. In this paper, the Boltzmann plot method was used to calculate the plasma temperature, and the formula is as follows:

(1) $$In\frac{I_{ki}\lambda_{ki}}{A_{ki}g_{k}}=-\frac{E_{k}}{kT}+In\frac{N_{o}hc}{g_{o}}$$

where *A_ki_* represents the transition probability of the electron from a high-energy level *k* to a low-energy level *I*, $\lambda_{ki}$ is the wavelength of spectral line, $I_{ki}$ is the measured relative spectral intensity, *N*_0_ is the number of particles in the ground state within a unit volume, and *g_k_* and *g*_0_ indicate the degeneracy of the high-energy level and ground state, respectively. *E_k_* is the excitation energy of the *k* level; *K* is the Boltzmann constant; *T* is the plasma excitation temperature; *h* and *c* are the Planck constant and the speed of light, respectively.

The spectral parameters for the calculation of the electron temperature are shown in Table 3.

The measurement of the electron density $N_{e}$ depends on Formula (2):

(2) $$\Delta \lambda_{1/2}=2\omega \frac{N_{e}}{10^{16}}$$

where $\Delta \lambda_{1/2}$ is the half-height width obtained by Lorentz fitting at appropriate points around the Fe I 426.047 nm spectral line and ω is the electron collision coefficient of the Fe I 426.047 nm line.

## 3. Results and Discussion

### 3.1. Morphology Characteristics of Laser-Induced Plasma during NGLW

**Figure 4:**
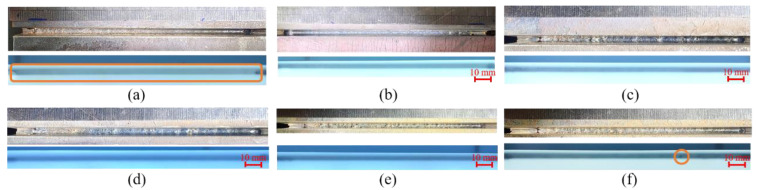
Weld morphology and X-ray inspection results of Experiment (**a**) No. 1, (**b**) No. 2, (**c**) No. 3, (**d**) No. 4, (**e**) No. 5, and (**f**) No. 6.

The test results of self-fusion welding are shown in Figure 4. Figure 4a–f successively represent the experiment results corresponding to the process parameters in Table 2. Each figure consists of two parts: the upper part is the weld, and the lower part is the corresponding X-ray test results. It is seen that only one porosity is found in Figure 4f, and no porosity defects were detected in the other welds. However, the overpenetration was obvious when the welding speed was 7 mm/s, and the sidewall was prone to being unfused when the welding speed was greater than 10 mm/s. The weld surface was easily oxidized when there was no shielding gas or the shielding gas flow was low. Therefore, in order to ensure the quality of subsequent wire feeding welding, the process parameters in No. 2 were selected for self-fusion welding.

**Figure 5:**
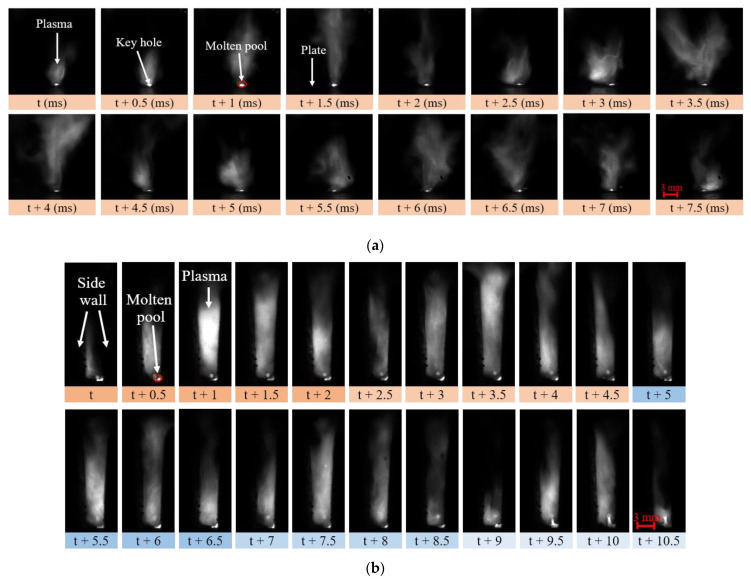
High-speed photography of laser-induced plasma: (**a**) butt plate laser welding; (**b**) narrow gap laser welding (defocus distance 10 mm, laser power 4.5 kW, welding speed 10 mm/s).

It was found that, even if the process parameters were identical, the plasma morphology and spectral intensity of narrow gap laser welding were quite different from that of butt plate laser welding (BPLW). As shown in Figure 5a, when the laser welding was carried out on a plate, the keyhole had a large opening, and the plasma eruption was periodic. During t ms to t + 2 ms, the volume of the plasma and flame increased and decreased successively. A new period of plasma eruption began at t + 2.5 ms and ended at t + 4.5 ms. A total of three periods of plasma eruption are shown in the figure, and the plasma eruption period was about 2 ms. In the process of narrow gap welding, the plasma eruption was constrained by the sidewall. It is seen from Figure 5b that the size of the plasma and flame depended on the groove shape. At the bottom of the image, the plasma eruption period is marked with different colors and times. Obviously, the periods varied dynamically within a certain range, with periods of about 2 ms for the first two groups and 1.5 ms for the last three groups. The vibration frequency of the observed laser-induced plasma was consistent with the results obtained by using the electrical sensor in the literature [22].

**Figure 6:**
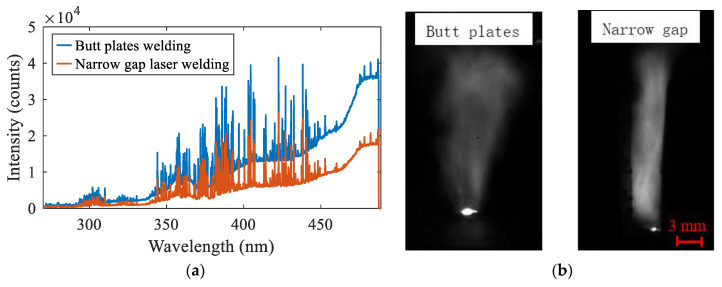
Plasma difference between plate laser welding and narrow gap laser welding: (**a**) spectrum; (**b**) image.

In addition, the difference in the spectral intensity between the two processes was compared, as shown in Figure 6. Since the material was consistent, the two types of welded joints had the same peak-profile under the same process parameters. The spectral intensity of narrow gap laser welding was smaller than that of plate self-fusion welding in the whole wave band. With the increase of the wavelength, the difference of the relative intensity of the continuous spectrum increased from 1000 to 19,000. As for why the intensity in narrow gap laser welding was lower, it is suggested that some electrons in the plasma were transferred to the metal on both sides of the groove. As a result, the number of electrons undergoing spontaneous transition was reduced and the light intensity was weakened.

### 3.2. Thermodynamic Characteristics of Laser-Induced Plasma during NGLW

**Figure 7:**
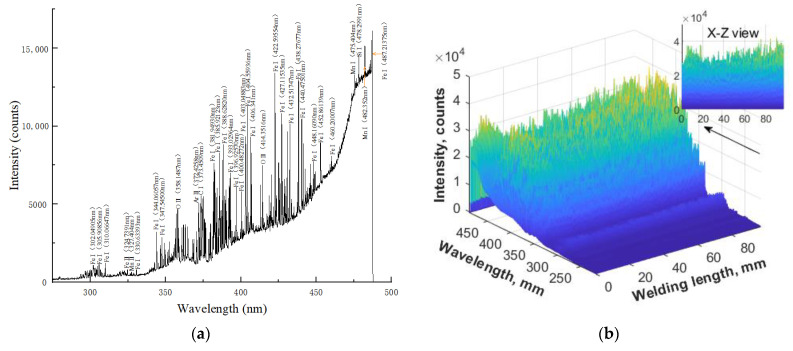
Spectrogram of self-fusion welding: (**a**) at a certain moment; (**b**) full welding process.

The spectral data collected at a certain time and in the whole narrow gap self-fusing laser welding process are shown in Figure 7a and Figure 7b, respectively. The line spectra were mainly distributed in the range of 350–450 nm. The line spectra in the range of 300–330 nm were mostly Fe I lines and a single Mn II line. In the range of 350–450 nm, there were many Fe I lines and a small number of Ar II, O II, and C I lines. The line spectra in the wavelength range of 460–490 nm were Fe I, Mn I, and Si I. The inset of Figure 7b represents the maximum light intensity value of all wavelengths during the welding process.

**Figure 8:**
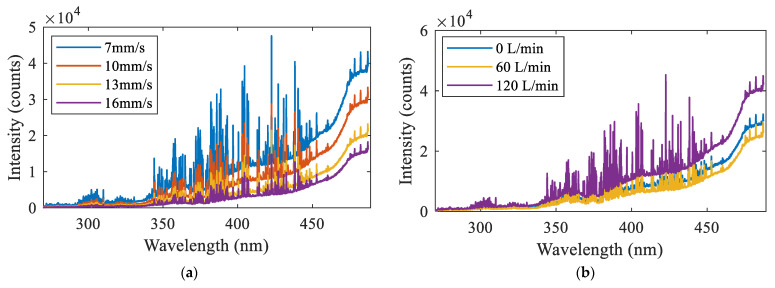
The influence of the process parameters on the spectral intensity: (**a**) welding speed; (**b**) shielding gas flow rate.

**Figure 9:**
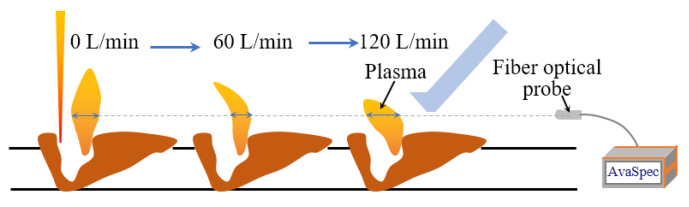
Influence of shielding gas flow rate on plasma morphology.

The influence of the process parameters on the spectral intensity is shown in Figure 8. It is seen from Figure 8a that the spectral intensity gradually decreased with the increase of the welding speed. This was because the increase of the welding speed reduced the heat input, resulting in the decrease of the number of excited particles $N_{k}$. According to the generation mechanism of the relative intensity, the spectral intensity decreased with the decrease of $N_{k}$. It is observed in Figure 8b that, with the increase of the flow rate of the shielding gas, the spectral intensity first decreased and then increased. It is suggested that the plasma shape changed under the action of the shielding gas, as shown in Figure 9. The optical fiber probe collected the intensity integral of the light at a certain height, and the relative light intensity was positively correlated with the size of the region emitting photons. When the gas flow rate increased from 0 L/min to 60 L/min, the plasma region at the acquisition height became smaller, resulting in the decrease of the light intensity. When the gas flow rate continued to increase to 120 L/min, the plasma height was greatly compressed, and the plasma region at the acquisition height increased significantly, resulting in a significant increase of the light intensity.

**Figure 10:**
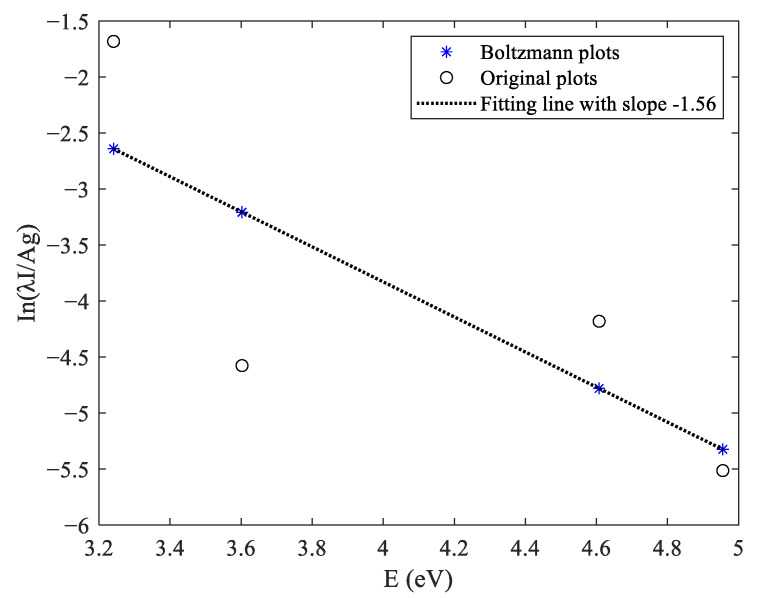
Electron temperature of Laser-induced plasma in narrow gap laser self-fusion welding.

**Figure 11:**
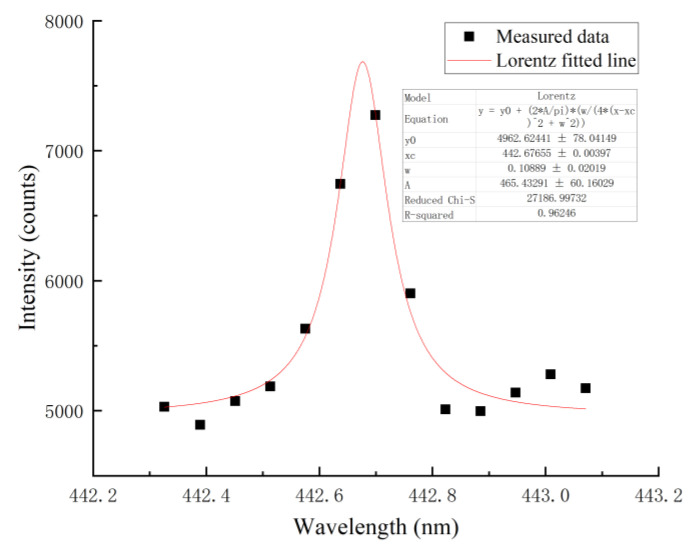
Measured Fe I 426.047 nm spectral line and the corresponding Lorentz fitting result.

In the normal self-fusion welding process, the calculated electron temperature was 7413.3 K, and the calculation process is shown in Figure 10. According to Formula (2), the electron density $N_{e}$ was 5.6714 × 10^15^ cm^−3^, as shown in Figure 11. According to the McWhirter criterion, the plasma generated during narrow gap laser self-fusion welding satisfied the local thermodynamic equilibrium state.

### 3.3. Relationship between Spectrum and Welding Quality

**Figure 12:**
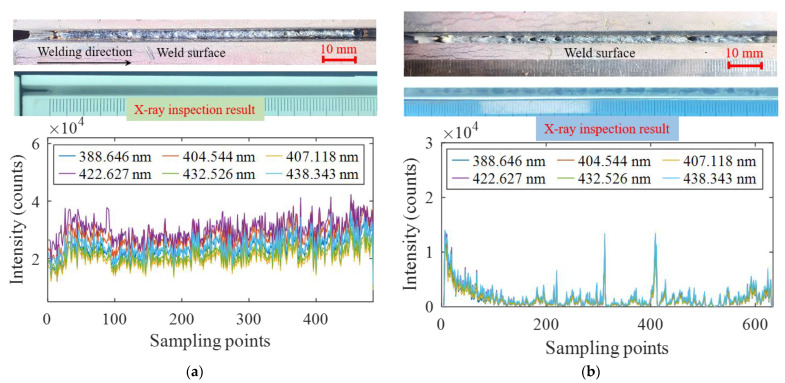
The relationship between the spectrum and weld quality during narrow gap laser self-fusion welding: (**a**) normal process; (**b**) water on base metal surface.

In order to explore the relationship between the characteristics of spectral intensity and weld quality, six Fe I spectral lines were selected to analyze the variation of the intensity in the process of laser self-fusion and laser wire filling welding. As shown in Figure 12a, when the base metal was in the normal state, the weld obtained by butt self-fusion welding had a well-formed surface without internal defects, and the spectral intensity of the Fe I spectral lines fluctuated between 15,000 and 35,000. When there was water on the base metal surface, surface porosity was observed on the weld. In addition, the X-ray inspection results showed that a large number of porosity defects with an irregular shape existed in the whole weld, as shown in Figure 12b. It is suggested that, when there was water on the base metal surface, the content of the hydrogen element in the molten pool increased greatly, which easily caused metallurgical porosity. Moreover, the water evaporation process absorbed heat, decreasing the temperature in the laser welding process and shortening the existence time of the liquid phase. This made it difficult for the metal plasma in the laser welding process to erupt out, aggravating the energy heterogeneity and increasing the probability of keyhole instantaneous instability. When the stability of the keyhole was affected, the keyhole was prone to collapse. After the collapse, the keyhole quickly closed with the flow of the molten pool to form bubbles and eventually evolved into pores. Under this condition, the measured intensity values of the Fe I spectral lines were less than 5000, which was attributed to the ejection behavior of the plasma caused by the unstable molten pool.

**Figure 13:**
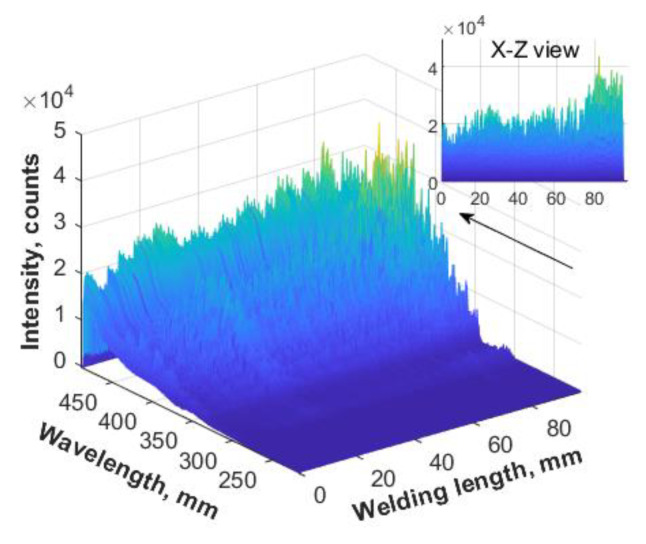
Three-dimensional spectra with water on the base metal surface.

**Figure 14:**
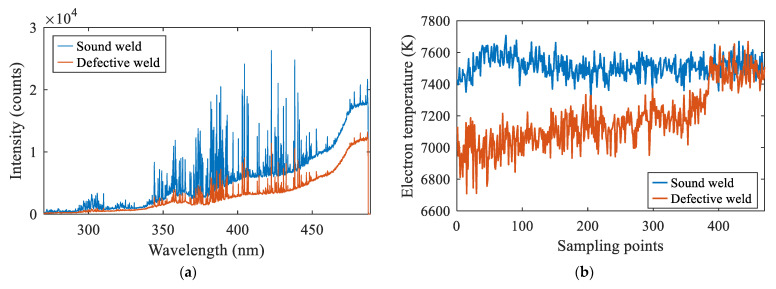
Spectral analysis of narrow gap laser self-fusion welding: (**a**) spectral intensity at a single moment; (**b**) electron temperature with time.

When there was water on the base metal surface, the collected three-dimensional spectra were as shown in Figure 13. From the X-Z view, it was seen that the spectral intensity value was smaller than that in the normal self-fusion welding. In order to compare the differences more clearly, the spectral intensity curves under the two conditions at a single moment are shown in Figure 14a. It was seen that the difference of the continuous spectrum increased from 200 to 6000 with the increase of the wavelength. The intensity difference of the line spectrum was 2000–4000 in the waveband of 270 nm–350 nm, and the optical intensity difference reached 15,000 in the waveband of 370–440 nm. In addition, the electron temperature was calculated under the two conditions, and the results are shown in Figure 14b. The electron temperature fluctuated in the range of 7400–7600 K under the normal process, while the value fluctuated in the range of 6900–7200 K when there was water on the base metal surface. The electron density with water on the base metal surface was 8.4193 × 10^15^ cm^−3^, which was greater than that in the normal welding condition.

**Figure 15:**
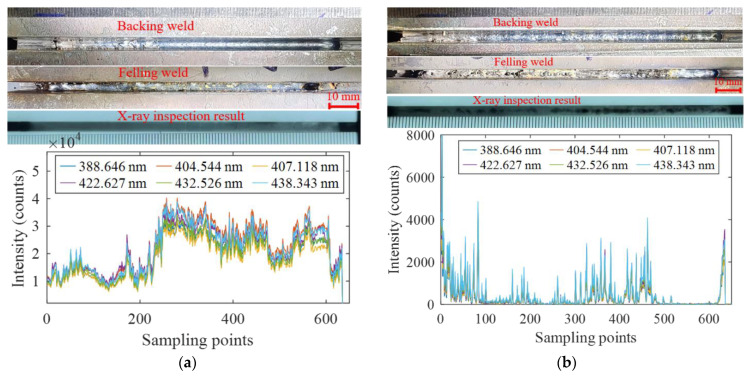

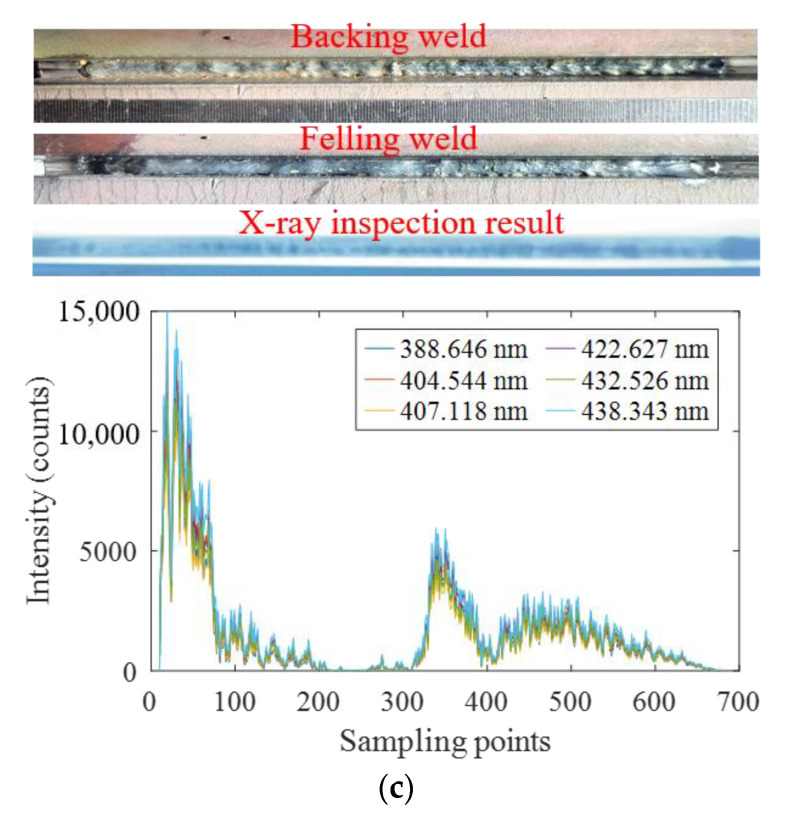
Experimental results of narrow gap laser wire filling welding: (**a**) normal process; (**b**) with water; (**c**) with oil on the weld surface.

The results of narrow gap laser wire filling welding are shown in Figure 15. As can be seen from Figure 15a, the weld surface was smooth in the normal welding condition, and there was no porosity defect inside. After the conduction of self-fusion welding, water and oil were present on the surface of the weld, respectively. The forming results are shown in Figure 15b and Figure 15c, respectively. It was seen that there were obvious pores on the weld surface after adding water, and the X-ray inspection results showed that there were dense pores inside. With oil preset on the weld, no obvious pores were found on the weld surface, but the X-ray inspection results showed that there were dense pores as well. The distribution of the spectral intensity was similar to that of self-fusion welding. The spectral intensity was strong under normal welding conditions, while the intensity decreased significantly when there were water and oil pollutants on the base metal surface.

## 4. Conclusions

This study investigated the thermomechanical properties of laser-induced plasma during narrow gap laser welding (NGLW) and realized the detection of porosity defects caused by pollutants using spectral diagnostic technology. The conclusions were as follows:

1. Under the same process conditions, the plasma morphology and spectral intensity of NGLW were different from that of butt plate laser welding (BPLW). The plasma was thin at the bottom and thick at the top during BPLW, while the plasma eruption was restricted by the sidewall in the NGLW process. The eruption periods of the plasma were almost the same under the two conditions. The measured relative spectral intensity was weaker in the NGLW process.
2. During narrow gap self-fusion welding, the spectral intensity decreased with the increase of the welding speed, while the intensity decreased first and then increased with the increase of the gas flow. Under the conditions of a 10 mm defocus distance and 10 mm/s welding speed, the electron temperature of the laser-induced plasma was 7413.3 K and the electron density was 5.6714 × 10^15^ cm^−3^, which accorded with the state of the local thermodynamic equilibrium.
3. When there was water on the groove surface, pores were generated on the weld surface during narrow gap laser self-fusion welding. A large number of dense pores were observed in the weld through X-ray detection. At this time, the relative spectral intensity in the whole waveband decreased and the electron temperature of the plasma decreased to 6900–7200 K. However, the electron density increased from 5.6714 × 10^15^ cm^−3^ to 8.4193 × 10^15^ cm^−3^.
4. During narrow gap laser wire filling welding, porosity defects were produced when there were water and oil pollutants on the surface of the last weld. The spectral intensity was significantly weakened compared to that collected in the normal welding process.

## Figures and Tables

**Table 1:** Chemical composition of base material (mass fraction, %).

	C	Mn	Cu	Si	S	P	Fe
A36	≤0.25	0.8~1.2	0.2	≤0.4	≤0.05	≤0.04	Balance

**Table 2:** Process parameters of narrow gap laser self-fusion welding.

No.	Welding Speed (mm/s)	Defocus Distance (mm)	Gas Flow Rate (L/min)
1	7	10	120
2	10	10	120
3	13	10	120
4	16	10	120
5	10	10	0
6	10	10	60

**Table 3:** The spectral parameters used for the calculation of the electron temperature.

Atom	Λ (nm)	*E_k_* (eV)	*g_k_*	*A_ki_* (*s*^−1^)
Fe I	393.015	3.241	7	1.99 × 10^6^
Fe I	344.078	3.603	7	1.71 × 10^7^
Fe I	396.914	4.608	7	2.26 × 10^7^
Fe I	310.055	4.956	7	1.35 × 10^7^

## Data Availability

All data generated or analyzed during this study are included in this published article.
